# Peak risk of SARS-CoV-2 infection within 5 s of face-to-face encounters: an observational/retrospective study

**DOI:** 10.1038/s41598-023-44967-x

**Published:** 2023-10-16

**Authors:** Takeshi Asai, Erina Kurosaki, Kaoru Kimachi, Masao Nakayama, Masaaki Koido, Sungchan Hong

**Affiliations:** 1 Faculty of Health and Sports Sciences, University of Tsukuba, Tennodai 1-1-1, Tsukuba, 305-8574 Japan; 2 Faculty of Physical Education, International Pacific University, Okayama, Japan

**Keywords:** Health care, Risk factors, Engineering

## Abstract

The link between aerosol dynamics and viral exposure risk is not fully understood, particularly during movement and face-to-face interactions. To investigate this, we employed Particle Trace Velocimetry with a laser sheet and a high-speed camera to measure microparticles from a human mannequin’s mouth. The average peak time in the non-ventilated condition (expiratory volume, 30 L; passing speed, 5 km/h) was 1.33 s (standard deviation = 0.32 s), while that in the ventilated condition was 1.38 s (standard deviation = 0.35 s). Our results showed that the peak of viral exposure risk was within 5 s during face-to-face encounters under both ventilated and non-ventilated conditions. Moreover, the risk of viral exposure greatly decreased in ventilated conditions compared to non-ventilated conditions. Based on these findings, considering a risk mitigation strategy for the duration of 5 s during face-to-face encounters is expected to significantly reduce the risk of virus exposure in airborne transmission.

## Introduction

From the early days of the coronavirus disease 2019 (COVID-19) pandemic to much of 2020, the World Health Organization (WHO) strongly believed that severe acute respiratory syndrome coronavirus 2 (SARS‑CoV‑2), the virus responsible for COVID-19, primarily spreads through larger respiratory droplets expelled by infected individuals while coughing, sneezing, or speaking^1–4^. However, reports of numerous airborne transmission possibilities have emerged, including outbreaks at the Wuhan hospital^5^ and a restaurant in Guangzhou^6–9^. In response to these findings, the WHO has now clearly identified two types of airborne transmission: transmission through short-range aerosols and transmission through long-range aerosols^10,11^. Furthermore, the Centers for Disease Control and Prevention (CDC) reported that the transmission of SARS-CoV-2 is associated with inhalation of airborne aerosol particles in poorly ventilated spaces^12–14^. Fluid dynamics have also played a role in classifying infections during the early stages of the pandemic. Infections caused by larger droplets (diameter of 5 µm or more) were classified as droplet infections, whereas those caused by smaller droplet nuclei (diameter of less than 5 µm) were classified as airborne infections^15,16^. However, recently, microparticles that have the property of immediately dropping to a surface (i.e., literally “droplets”) have been classified as having a diameter of 100 µm or more^17^ and particles with a smaller diameter have been classified as aerosols^18^. Droplets with a diameter of 100 µm or more drop to the ground within 3–5 s; however, large aerosols with a diameter of 5–100 µm float in the air for several minutes to several dozens of minutes, and even smaller aerosols with a diameter of less than 5 µm float in the air for several hours^3,19,20^.

Therefore, the risk of infection from aerosols, which have a long residence time in the air, is thought to be greater than the risk of infection from droplets, which have a short residence time in the air^13^. Moreover, airborne infection is highly likely to be the primary infection route of SARS-CoV-2^21,22^. The behavior of airborne aerosols that cause airborne infections^23,24^ is extremely complex and has many unclear aspects due to it being affected by mainstream and ventilation flow fields^25–27^. Therefore, the relationship between the dynamics of aerosol particles suspended in the air and the risk of viral exposure needs to be clarified^21,28–30^. Furthermore, as behavioral restrictions due to COVID-19 have been recently relaxed and social activities have resumed, human movement and traffic have become more active^31,32^. Hence, visualization of aerosol particles derived from human exhalation (jet flow) during movement or exercise and analysis and evaluation of viral exposure risk are very important^33–41^.

Therefore, this study aimed to visualize and measure the flow field of aerosols derived from exhalation (jet flow) during face-to-face encounters with another person and to clarify the viral exposure risk when passing by another person. We employed a full-scale mobile mannequin and a Particle Tracking Velocimetry (PTV) system in what is considered the world’s first such attempt. Furthermore, we compared the behavior of the flow fields of aerosols during face-to-face encounters under ventilated versus non-ventilated conditions to assess the aerodynamic characteristics of ventilation and its impacts on the viral exposure risk.

## Results

### Time-series change in the microparticle count under non-ventilated and ventilated conditions

**Figure 1:**
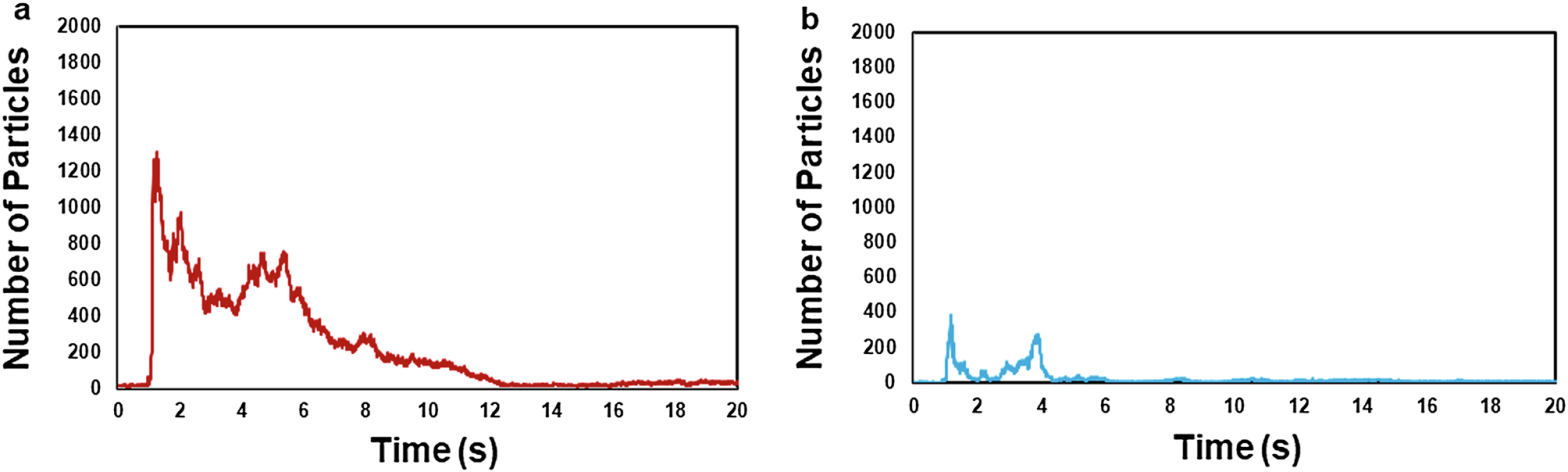
An illustration of microparticle counts (viral exposure risk index) in the non-ventilated condition (**a**) and the ventilated condition (**b**), with an expiratory volume of 30 L and a passing speed of 5 km/h.

The microparticle count (viral exposure risk index) in the non-ventilated condition (expiratory volume, 30 L; passing speed, 5 km/h) peaked within approximately 5 s (~ 1.24 s) immediately following face-to-face encounters. The average peak time in the non-ventilated conditions was 1.33 s (S.D. = 0.32 s) and that in the ventilated conditions was 1.38 s (S.D. = 0.35 s). Subsequently, the microparticle count decreased (Fig. 1a). Similarly, the microparticle count in the ventilated condition (30 L; 5 km/h) peaked within approximately 5 s (~ 1.17 s) and decreased thereafter (Fig. 1b). The peak microparticle count in the non-ventilated condition (30 L; 5 km/h) was 1306 particles, which was greater than the peak count of 387 particles in the ventilated condition. A similar trend was evident across other expiratory volumes (55 L, 80 L) and passing speeds (10 km/h, 15 km/h, 20 km/h) in both the non-ventilated and ventilated conditions. The average microparticle count at its peak in the non-ventilated condition was significantly higher than that in the ventilated condition (p = 0.001, after adjustment using the Bonferroni correction).

### Characteristics of average microparticle count curves for non-ventilated and ventilated conditions

**Figure 2:**
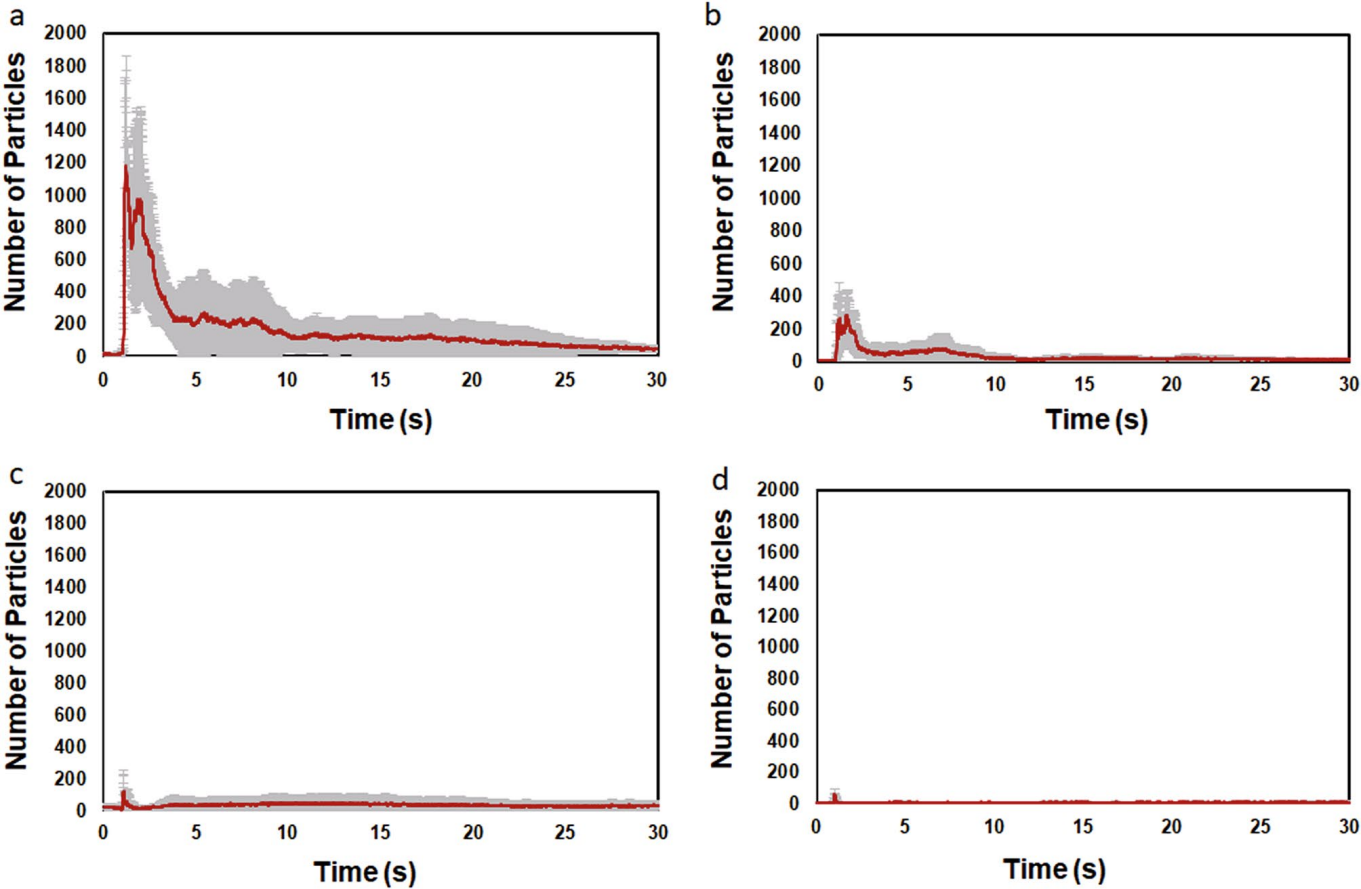
The average microparticle count for an expiratory volume of 30 L under non-ventilated conditions following face-to-face encounters at various passing speeds of 5 km/h (**a**), 10 km/h (**b**), 15 km/h (**c**), and 20 km/h (**d**). In all instances, the average microparticle count peaks within approximately the first 5 s after face-to-face encounters and then sharply decreases.

The average microparticle count in the case of an expiratory volume of 30 L under the non-ventilated condition peaked within approximately 5 s in the initial period after face-to-face encounters in all passing speed cases of 5 km/h, 10 km/h, 15 km/h, and 20 km/h, after which it decreased sharply (Fig. 2a–d). The average microparticle count approximately 30 s after face-to-face encounters decreased to the same level before passing (~ 50 particles). As the passing speed decreased, the average microparticle count also tended to increase.

**Figure 3:**
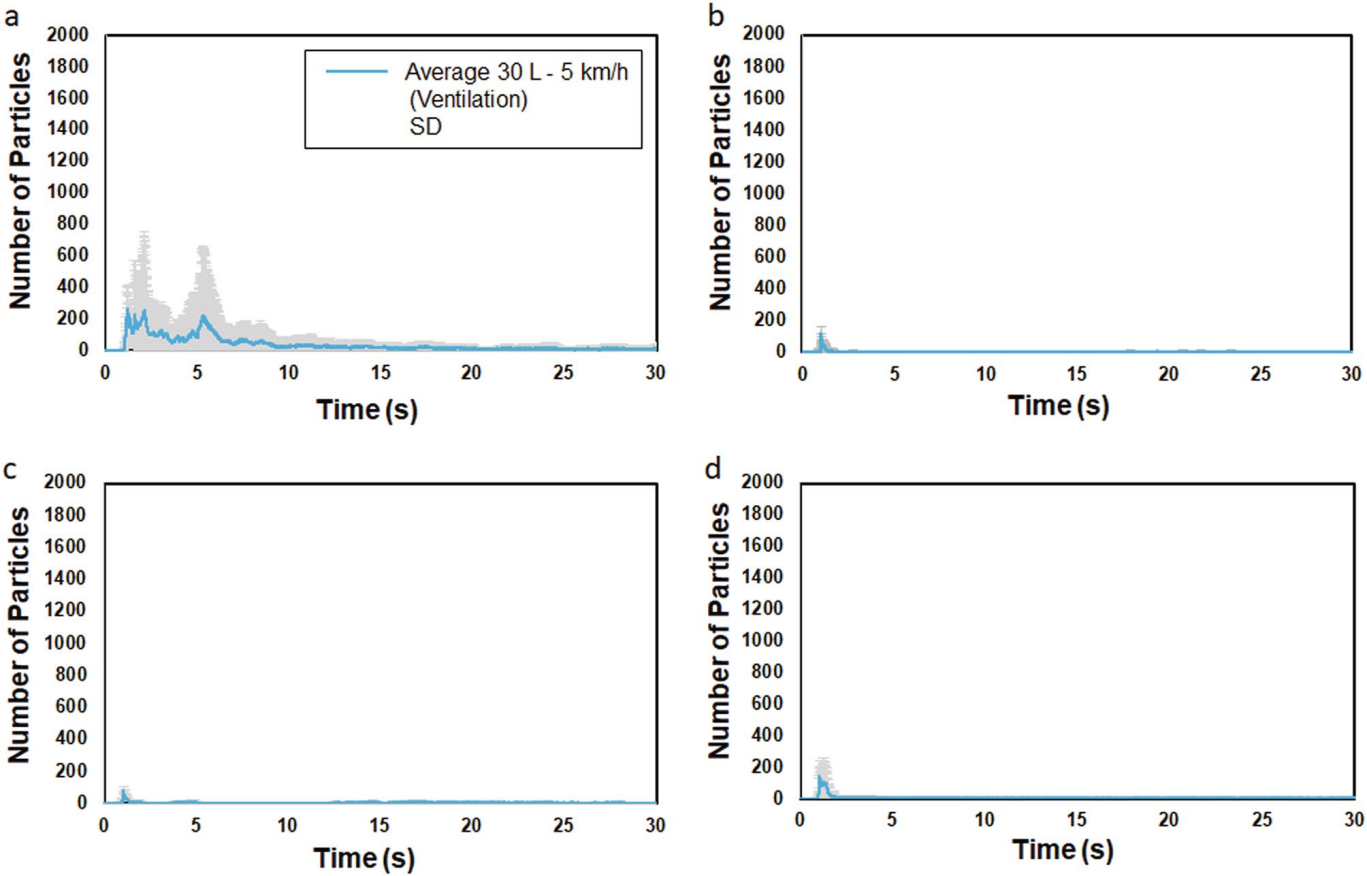
The average microparticle count for an expiratory volume of 30 L under ventilated conditions following face-to-face encounters at various passing speeds of 5 km/h (**a**), 10 km/h (**b**), 15 km/h (**c**), and 20 km/h (**d**). In all instances, the average microparticle count peaks within approximately the first 5 s after face-to-face encounters and then sharply decreases.

Further, the average microparticle count in the case of an expiratory volume of 30 L under ventilated conditions peaked within approximately 5 s in the initial period after face-to-face encounters, after which it decreased sharply (Fig. 3a–d). The average microparticle count in the case of passing speed of 5 km/h tended to be larger than the count in the case of other passing speeds of 10 km/h, 15 km/h, and 20 km/h.

### Average microparticle count at peak and after 10 s, 20 s, and 30 s

**Figure 4:**
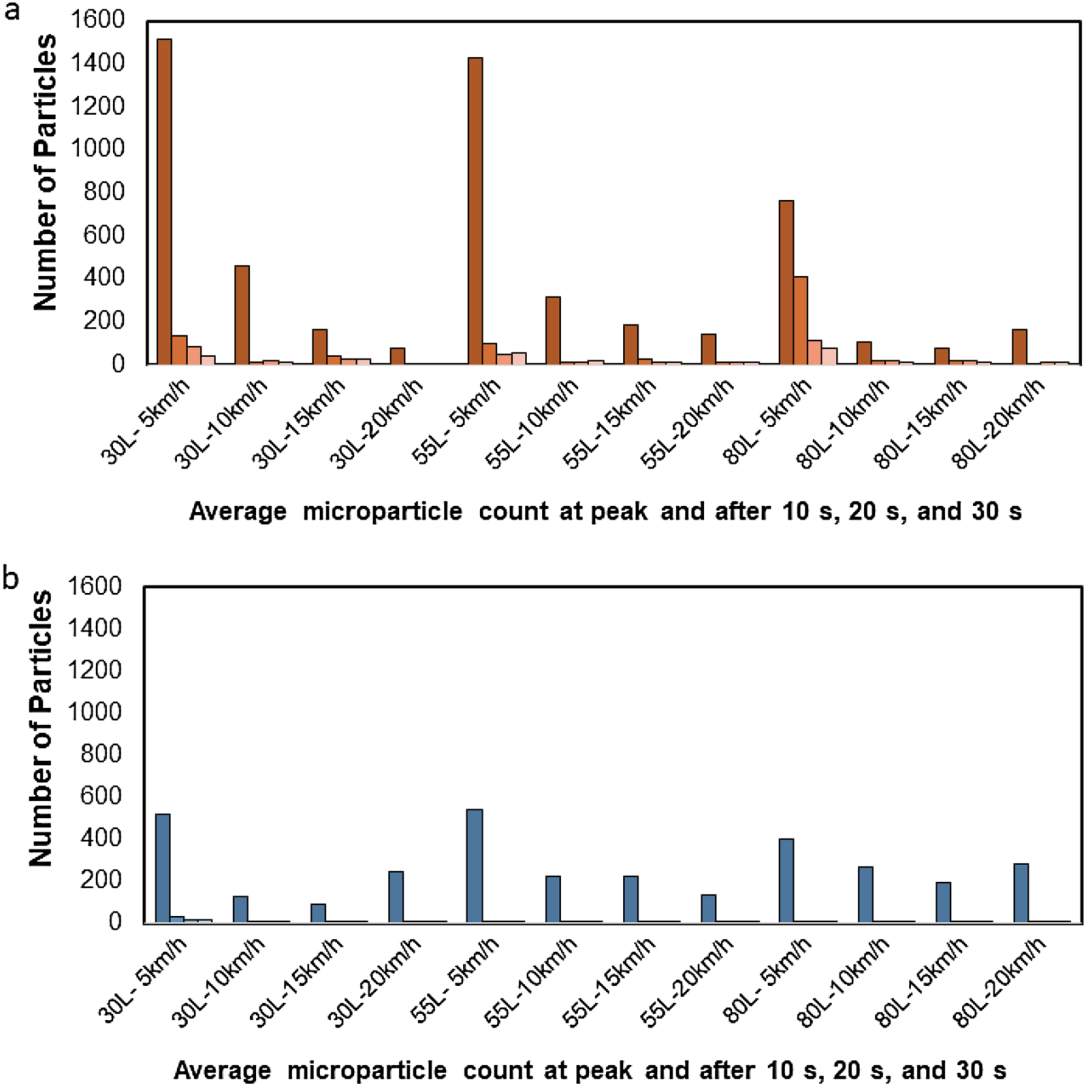
The average microparticle count at peak and after 10 s, 20 s, and 30 s for expiratory volumes of 30 L, 55 L, and 80 L under non-ventilated (**a**) and ventilated (**b**) conditions. For the passing speeds that increased from 10 to 15 to 20 km/h the average microparticle count for each speed tends to decrease over time, similar to the trend observed at 5 km/h.

The average microparticle count for an expiratory volume of 30 L and passing speed of 5 km/h in the non-ventilated condition was 1516 particles (standard deviation (S.D.) = 576 particles) at peak, 133 (S.D. = 97) after 10 s, 88 (S.D. = 90) after 20 s, and 42 (S.D. = 23) after 30 s, with results showing that the count decreased with time (Fig. 4a). Further, for the passing speeds that increased from 10 to 15 to 20 km/h, the average microparticle count for each speed tended to decrease over time, as in the case of passing speed of 5 km/h. Multiple comparisons of the average microparticle count at peak in the non-ventilated condition using the Bonferroni correction showed that for an expiratory volume of 30 L, the passing speed of 5 km/h had a significantly larger peak than the passing speeds of 10 km/h (p = 0.134 × 10^–9^), 15 km/h (p = 0.185 × 10^–12^), and 20 km/h (p = 0.342 × 10^–13^). Furthermore, for the other expiratory volumes of 55 L and 80 L, the passing speed of 5 km/h had a significantly larger peak than the other passing speeds. The average microparticle count in the case of an expiratory volume of 30 L and passing speed of 5 km/h in the ventilated condition was 519 particles (S.D. ≥ 383 particles) at peak, 31 (S.D. = 45) after 10 s, 15 (S.D. = 11) after 20 s, and 18 (S.D. = 21) after 30 s, with the results showing that the count decreased over time, as in the non-ventilated condition (Fig. 4b). Moreover, in the cases where the passing speeds increased from 10 to 15 to 20 km/h, the average microparticle count in each case tended to decrease over time. Comparisons of only the peak value of each average microparticle count in the ventilated condition showed that for any expiratory volume case, the value for the passing speed of 5 km/h was larger than the value for other passing speeds of 10 km/h, 15 km/h, and 20 km/h cases; however, the difference was not as significant as that for the non-ventilated condition.

### Dependence of the average peak microparticle count (curve) on the passing velocities and expiratory volumes

The relationship between the passing speed and average microparticle count at the peak in the non-ventilated condition roughly showed that a larger passing speed tended to reduce the microparticle count (R =  − 0.79, p = 0.0077). In contrast, the average microparticle count at peak in the ventilated condition was the highest for the passing speed of 5 km/h, regardless of the expiratory volume; however, it was unclear if this finding depended on the passing speed.

The relationship between the expiratory volume and the average microparticle count at peak in the non-ventilated condition roughly showed that a larger expiratory volume tended to slightly decrease the microparticle count. However, the average microparticle count at peak in the ventilated condition did not show any significant dependency on the expiratory volume.

## Discussion

The jet flow containing microparticles ejected from the mouth of a moving mannequin (exhalation-derived particle model) forms a large-scale turbulent wake vortex structure that is centered on the back of the head and back, spreading over a wide area (Supplementary Fig. 1). The formation and diffusion of large-scale turbulent wake vortex structures is a typical flow structure of moving objects (bluff bodies) in fluids^42^. Moreover, the turbulent vortex structure behind the mannequin in this study was thought to exhibit a similar unsteady vortex structure. In the context of the current airflow patterns, our findings demonstrate that the peak risk of viral exposure occurs within a 5-s window during face-to-face interactions, irrespective of whether ventilation is adequate or lacking.

When coughing without a mask, microdroplets quickly fall within 2 m when there is no wind; however, previous studies have reported that the microdroplets float in an area of 6 m or more from the outlet when there is a tailwind of 4 km/h or 15 km/h^13,43,29^. In the present study, most of the particles ejected during movement were thought to be floating in an area of at least 6 m or more from the outlet, despite the mainstream being on the opposite side. A previous study has reported that the droplet count emitted in non-ventilated environments by a stationary human ranged from 947 to 2085 particles when coughing and 112 to 6720 during vocalization^44^. The peak droplet count during exercise (30 L, 5 km/h) that was obtained in this study was 1516 particles, which was close to the droplet count emitted when coughing in a stationary position.

Droplets and aerosols (including microdroplets) are known to have different gravitational falling speeds depending on their particle size and water content^22,45,46^. Studies have shown that for gravitational falling speeds under normal conditions and in a no-wind environment, the elapsed time to fall to the ground from mouth height (~ 1.5 m) was approximately 3–5 s at a particle size of 100 µm, 6 s for 10 µm, and 33 s for 5 µm, with the possibility of a particle moving over 1 m from the infected individual in the 100 µm case^13,19^. The size of the nylon microparticles used in this study was 6–14 µm, which was larger than the general aerosols emitted from exhalation (< 5 µm); however, these particles were speculated to float for about 6 min under no-wind conditions. Moreover, it is thought that the unsteady exhalation flow field and the dynamics of microdroplets and aerosols during exercise can be reasonably studied with these particles^47,48^.

The peak microparticle count at walking speed (5 km/h) under the non-ventilated condition was larger than that at the jogging (10 km/h), running (15 km/h), and sprinting (20 km/h) speeds under all exhalation conditions, with a peak observed within 5 s of face-to-face encounters, and a subsequent tendency to sharply decrease. One of the main reasons for this is that the microparticle count rapidly decreased due to the diffusion of turbulent wake eddies that formed behind the mannequin after the mainstream of the exhalation (jet) had passed^49^.

The experimental result of “a peak observed within 5 s of face-to-face encounters” should be interpreted in the context of this specific experiment. It may not necessarily apply to all breathing activities and should be approached with caution, as it can be influenced by factors such as coughing, sneezing, symptoms, and individual variability.

Furthermore, the higher passing speed cases tended to decrease the microparticle count. It was speculated that a higher passing speed case increased the relative speed between the jet and outer stream, thereby promoting diffusion. Based on these results, we considered that the risk of viral exposure was highest within 5 s of face-to-face encounters for any speed case, and higher passing speeds were thought to reduce the risk of viral exposure because the relative speed increased and the diffusion of microparticles was promoted.

Under the ventilated condition, the peak microparticle count at walking speed (5 km/h) was larger than that at the other passing speed cases; however, it was approximately 55% less than that in the non-ventilated condition. Moreover, the microparticle count sharply decreased after the peak and remained at a low value of about 50 particles or fewer. This was thought to be due to the fact that controlled ventilation was present in the ventilated condition in this study, which promoted microparticle diffusion, decreasing the dependency of the peak microparticle count on the passing speed and sharply decreasing the microparticle count after the peak^29,50^. Based on these results, we considered that the risk of viral exposure was highest within 5 s after face-to-face encounters, even for the ventilated condition; however, the exposure risk after passing greatly decreased relative to that in the non-ventilated condition due to the diffusion effect of ventilation.

From the above, we considered that the risk of viral exposure during face-to-face encounters while walking or jogging peaks within 5 s of passing and that the risk subsequently sharply decreases due to diffusion of the wake. It has been reported that the cough droplet count that is deposited during face-to-face encounters was the highest at 60 cm in front of the cough outlet and 30 cm below it, with at least 90% of the droplets being deposited within 90 cm of the front of the outlet^41,51^. Therefore, during face-to-face encounters, we believe the risk of viral exposure will greatly decrease by hedging against the risk during the 5-s period and moving one’s course laterally to the direction of travel, passing through the windward side, interrupting inhalation, and leaving a physical distance of at least 1 m.

Additionally, even in ventilated cases, cases of infection have been reported in crowded places such as large outdoor events^51^. Therefore, hedging against the risk during the 5-s period face-to-face encounters is effective. When exercising outdoors, implementing this risk-hedging behavior may greatly reduce the risk of viral exposure.

Our findings indicate that the peak risk of viral exposure occurs within a 5-s window during face-to-face interactions, under both ventilated and non-ventilated conditions. Additionally, after the initial 5-s window of face-to-face interaction, the risk of viral exposure significantly decreases in ventilated settings compared to non-ventilated ones. Therefore, taking precautionary measures during these initial 5 s will greatly reduce the risk of airborne viral exposure. The insights gained from this study could be applicable to reducing infection risks in cases mediated by aerosols, such as influenza and monkeypox. In the future, it will be crucial to explore not only the risks of associated with face-to-face interactions but also those occurring during various forms of physical activity, sports competitions, and sporting events.

In conclusion, our study has revealed several key findings. The highest risk of viral exposure during face-to-face interactions occurs within a 5-s window, regardless of ventilation conditions. In non-ventilated conditions, the highest microparticle count was observed at a walking speed of 5 km/h; however, this count rapidly decreased after the initial 5-s window due to the diffusion of turbulent wake eddies formed by the exhalation jet. Higher passing speeds during face-to-face encounters tended to decrease the microparticle count, likely because of the increased relative speed between the exhalation jet and surrounding air, thereby promoting microparticle diffusion. Controlled ventilation significantly reduced the microparticle count compared to non-ventilated conditions, with a sharp decrease observed after the peak. This suggests that ventilation enhances microparticle diffusion and reduces the reliance on passing speed to determine the peak particle count. To reduce the risk of viral exposure during face-to-face encounters, individuals can adopt preventive measures, such as changing their direction to the side opposite the wind, interrupting their inhalation, and maintaining a physical distance of at least 1 m. These actions are particularly effective during the critical 5-s interaction period. Based on our findings, this study has implications for reducing aerosol-mediated transmission of various pathogens, such as SARS‑CoV‑2, influenza, and monkeypox.

However, the limitations of this study should be acknowledged. We estimated the risk of viral exposure based on microparticle exposure; therefore, future research should consider additional factors, such as air concentration and virus deposition on surfaces, to improve risk assessment accuracy^52–54^. Additionally, our measurements were conducted in 2D, and more detailed analyses using 3D measurements may be beneficial^55,56^.

Future research should explore the risks associated with different forms of physical activity, including sports competition, to enhance the current understanding of airborne viral transmission.

## Methods

### Ethics

This study did not require approval from an Institutional Review Board, in accordance with research governance guidelines, as it utilized a human mannequin rather than human participants.

### Human mannequins, the motorized cart, and the microparticle ejection device

**Figure 5:**
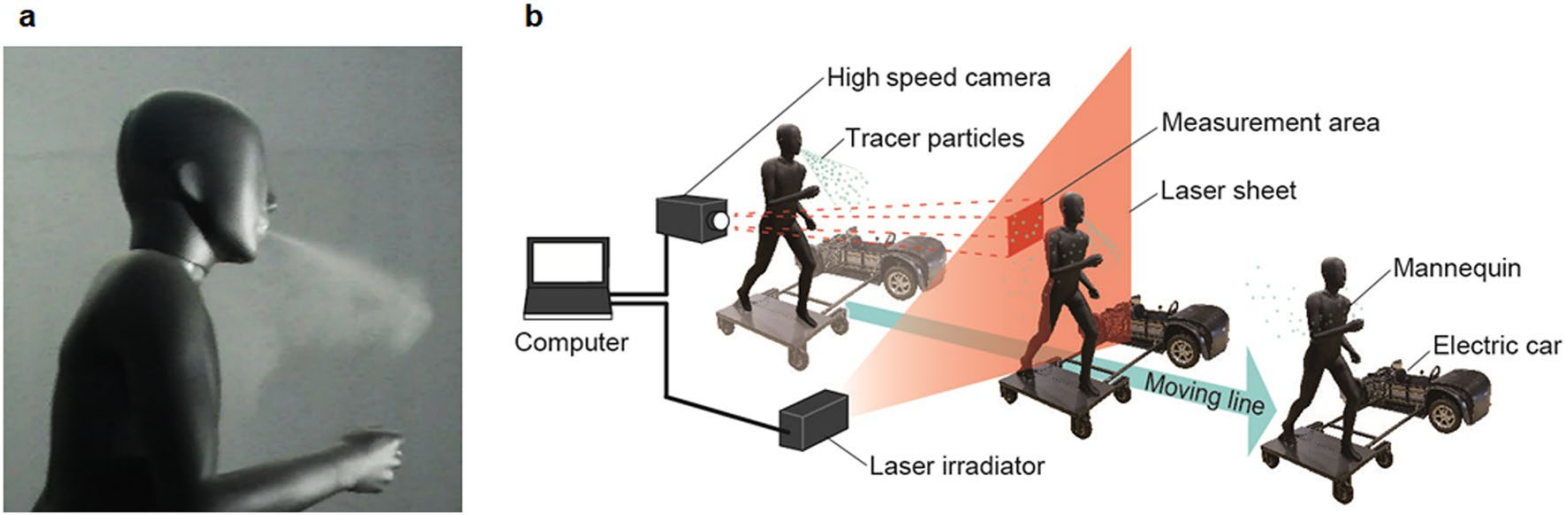
Built-in microparticle blowout device (**a**) and the experimental setup using the particle trace velocimetry system (**b**), which includes a laser sheet (depicted in red) and a high-speed camera.

On the side of an electric cart (Saitou Craft Co. Ltd., Japan) measuring 2 m, a full-scale human mannequin (height = 1700 mm) made of Fiber Reinforced Plastics with a microparticle ejection device [Solid Particle Disperser Series of RBG 1000 (PALAS Gmbh, Germany)] connected to the outlet was set, to achieve translational motion of a mannequin accompanied by a jet stream (Fig. 5). The microparticle ejection device comprised a rotating brush for generating tracer particles from the packed microparticles (particle size, 6–14 µm; mean, 10 µm; KATO KOKEN Co., Ltd., Japan, nylon material) and a compressor that generated a jet stream. In this study, the amount of microparticles supplied per unit of time was kept constant (~ 2.28 × 10^8^ particles), and the expiratory volume condition was determined by the compressor air volume.

The microparticle size used in this study was smaller than that of droplets (> 100 µm) and larger than that of small aerosols (< 5 µm). The average size of the aerosols (microdroplets) in this experiment was 10 µm^10,13^ which was the smallest size measurable by this experimental setup. These aerosols served as the model for the experiment, and their residence time of approximately 6 min was significantly longer than the actual experimental duration of 1 min.

### Microparticle measurement system

The visualization and measurement of microparticles ejected from the mouth of the human mannequin^57,58^ were conducted using a particle tracking velocimetry (PTV) method that comprised a laser sheet (red) (LMD-MO-058, KATO KOKEN, Japan) and a high-speed camera (K9 High-speed Camera, KATO KOKEN Co. Ltd., Japan)^59,60^. The microparticle count measurement area was a two-dimensional plane measuring 0.6 × 0.6 m that was set 0.3 m from the right side of the head of the mannequin. Recordings were made for 60 s at a frame rate of 30 frames per second (fps) and resolution of 1280 × 1028 dps after the ear of the mannequin passed the laser sheet. The microparticles were counted using a PTV system, which measured the quantity of microparticles at a rate of 30 fps.

Based on multiple images obtained from the video footage, we used image processing techniques to track the positions of individual microparticles over time. Subsequently, we calculated parameters such as particle count.

The highest count observed was designated as the “peak microparticle count”. Additionally, the time at which this peak occurred was defined as the “peak time”, and the average value of this peak time across each of the five trials was referred to as “the average peak time”. Furthermore, the average microparticle count was determined by calculating the mean value for each of the five trial, and the peak value of this average was referred to as the “average microparticle count at peak”.

### Experimental conditions and analysis methods

The human mannequin moved straight over a measurable distance of approximately 20 m, and the measurement area was set in the position 10 m from the start, where the mannequin acceleration and deceleration impacts were considered small. The passing speed conditions of the jet stream mannequin were set to correspond to typical movement speeds: 5 km/h for walking, 10 km/h for jogging, 15 km/h for running, and 20 km/h for sprinting^61^.

To simulate the respiratory volumes during typical physical activities, such as walking, jogging, and running, we set the jet flows (ventilation per minute) at 30 L/min, 55 L/min, and 80 L/min, respectively^62–65^. In this experiment, we used a relatively consistent jet discharge without abrupt bursts of air resembling coughing. The microparticle count was measured five times under each trial condition, and the average of those measurements was set as the average microparticle count.

For the non-ventilated condition, each trial was conducted in a small gymnasium (8 m high × 6 m wide × 30 m deep); all the openings (4 m wide × 5 m high) in front and behind the direction of movement of the mannequin were closed, and the ventilation equipment stopped. After the measurement in each trial, the openings were opened to promote ventilation with external air, and the microparticles that fell to the ground were wiped off. The indoor temperature was 10 °C ± 5 °C, and the relative humidity was 50% ± 10%.

For the ventilated condition, all the openings of the small gymnasium (8 m high × 6 m wide × 30 m deep) were opened, and a rotational flow of approximately 1.0 m/s was blown by a large fan from 8 m in front of the mannequin in the direction of movement of the measurement laser sheet. After the measurement in each trial, the microparticles that fell to the ground were wiped off in the same manner as with the non-ventilated condition.

### Statistical analysis

Multiple comparison tests based on the Bonferroni correction were conducted for the average microparticle counts at peak and after 10, 20, and 30 s for each trial, using the movement speed and expiratory volume as factors (BellCurve for Excel ver. 4.01, Social Survey Research Information Ltd.). Pearson’s correlation coefficient was used to determine the correlations.

### Supplementary Information

Supplementary Figure 1.

## Data Availability

The data that support the findings of this study are available upon request from T.A. (the corresponding authors).
