# Supplementary material for: Peak risk of SARS-CoV-2 infection within 5 s of face-to-face encounters: an observational/retrospective study
Source: Sci Rep. 2023 Oct 16;13:17520. doi: 10.1038/s41598-023-44967-x (PMC10579401; doi:10.1038/s41598-023-44967-x)
Supplement: Supplementary file 1 — Supplementary Figure 1. [file 41598_2023_44967_MOESM1_ESM.docx]

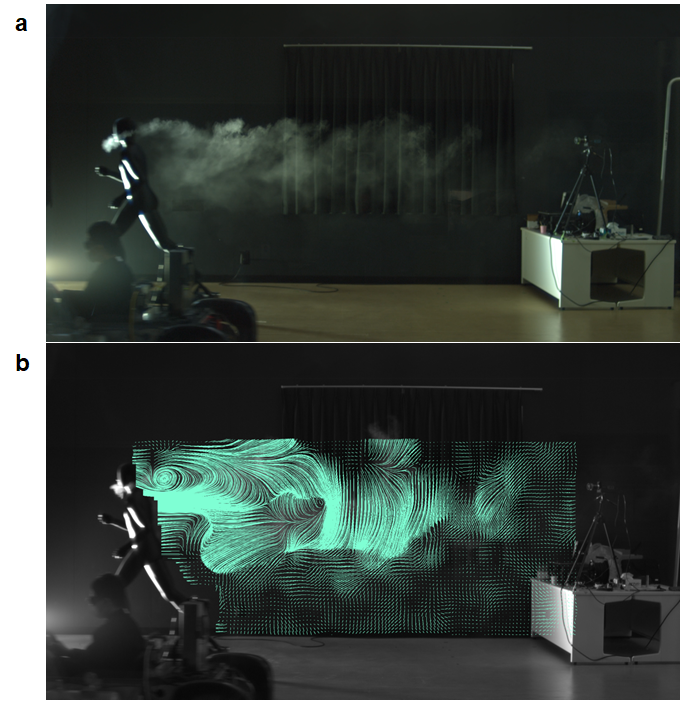


Supplementary Fig. 1

Examples of a snapshot from a high-speed video image (a) and a streamline of jet flow (b) containing microparticles expelled from the mouth of a moving mannequin (exhalation-derived particle model), demonstrating the formation of a large-scale turbulent wake vortex structure centered on the back of the head and extending over a wide area.
